# Re﻿placing dietary animal-source proteins with plant-source proteins changes dietary intake and status of vitamins and minerals in healthy adults: a 12-week randomized controlled trial

**DOI:** 10.1007/s00394-021-02729-3

**Published:** 2021-11-27

**Authors:** Tiina Pellinen, Essi Päivärinta, Jarkko Isotalo, Mikko Lehtovirta, Suvi T. Itkonen, Liisa Korkalo, Maijaliisa Erkkola, Anne-Maria Pajari

**Affiliations:** 1 Department of Food and Nutrition, University of Helsinki, Helsinki, Finland; 2 The Computing Sciences Unit, Tampere University, Tampere, Finland; 3 Institute for Molecular Medicine Finland, University of Helsinki, Helsinki, Finland

**Keywords:** Animal-based foods, Plant-based foods, Flexitarian diet, Group B vitamins, Iron status, Iodine status

## Abstract

**Purpose:**

A shift towards more plant-based diets promotes both health and sustainability. However, controlled trials addressing the nutritional effects of replacing animal proteins with plant proteins are lacking. We examined the effects of partly replacing animal proteins with plant proteins on critical vitamin and mineral intake and statuses in healthy adults using a whole-diet approach.

**Methods:**

Volunteers aged 20–69 years (107 female, 29 male) were randomly allocated into one of three 12-week intervention groups with different dietary protein compositions: ANIMAL: 70% animal-source protein/30% plant-source protein; 50/50: 50% animal/50% plant; PLANT: 30% animal/70% plant; all with designed protein intake of 17 E%. We analysed vitamin B-12, iodine, iron, folate, and zinc intakes from 4-day food records, haemoglobin, ferritin, transferrin receptor, folate, and holotranscobalamin II from fasting blood samples, and iodine from 24-h urine.

**Results:**

At the end point, vitamin B-12 intake and status were lower in PLANT than in 50/50 or ANIMAL groups (*P* ≤ 0.007 for all). Vitamin B-12 intake was also lower in 50/50 than in ANIMAL (*P* < 0.001). Iodine intake and status were lower in both 50/50 and PLANT than in ANIMAL (*P* ≤ 0.002 for all). Iron and folate intakes were higher in PLANT than in ANIMAL (*P* < 0.001, *P* = 0.047), but no significant differences emerged in the respective biomarkers.

**Conclusions:**

Partial replacement of animal protein foods with plant protein foods led to marked decreases in the intake and status of vitamin B-12 and iodine. No changes in iron status were seen. More attention needs to be paid to adequate micronutrient intakes when following flexitarian diets.

**Clinical trial registry:**

NCT03206827; registration date: 2017–06-30.

**Supplementary Information:**

The online version contains supplementary material available at 10.1007/s00394-021-02729-3.

## Introduction

An urgent need exists to create both an environmentally and nutritionally sustainable food system. To decrease the environmental effects of food production, it is essential to reduce consumption of animal-derived foods such as ruminant meat and dairy. In current Western diets, the consumption of these foods is excessive also relative to nutritional recommendations [1, 2]. Shifting toward plant-based flexitarian diets and replacing animal protein sources with plant protein ones would likely improve health and reduce the risk of mortality, cardiovascular diseases, colorectal cancer, and type 2 diabetes [3–6]. The number of flexitarian consumers who abstain from eating meat regularly is on the rise [7].

Cross-sectional studies have reported that strictly plant-based diets are characterized by several nutritional benefits such as high folate intake and status [8–10]. However, they may also pose some nutritional risks, such as low intake and status of some critical nutrients, compared with omnivorous mixed diets. Lower vitamin B-12 and iodine intake and status and a risk for deficiency have been reported not only in vegan [8–12], but also to some extent in vegetarian diets [9–13]. Lower vitamin B-12 intake and status in a vegan diet have also been noted in a 4-week randomized clinical trial [14], and lower iodine excretion in subjects with a lactovegetarian diet in a short-term (48 h) controlled experimental study [15]. Cross-sectional studies have also shown lower zinc intakes among vegans and vegetarians [8, 10, 12].

In cross-sectional studies, iron intake in plant-based diets has been reported to be similar or higher than in omnivorous diets [8, 10, 16, 17]. Despite higher intakes, vegetarians in some [8, 17], but not all [18–20], studies have had lower iron stores than their non-vegetarian peers. This may be due to lower bioavailability of non-heme iron in plant foods than of heme iron in animal-derived foods. Typically, heme iron contributes 10–15% of iron intake in omnivorous populations [21]. Plant-based foods, including whole grains, legumes, seeds, and nuts, also contain numerous anti-nutrients, such as phytates that are known to decrease iron absorption in the small intestine. On the other hand, plant foods typically contain vitamin C, an enhancer of the bioavailability of non-heme iron [22].

The composition of the diet as a whole is critical to the bioavailability, and thus, to the status of vitamin B-12, folate, iron, zinc, and iodine. It is important to investigate in a controlled setting the extent to which animal protein sources can be replaced by plant protein sources in flexitarian, plant-based diets to achieve health advantages, while simultaneously ensuring adequate intake of critical vitamins and minerals. Here, we report the results of a 12-week randomized controlled trial with a parallel design using a whole-diet approach to examine the effects of partial replacement of animal proteins with plant proteins on the intakes of folate, vitamin B-12_,_ iron, zinc, and iodine and their nutritional status biomarkers among healthy adults.

## Subjects and methods

### Study design and subjects

**Fig. 1:**
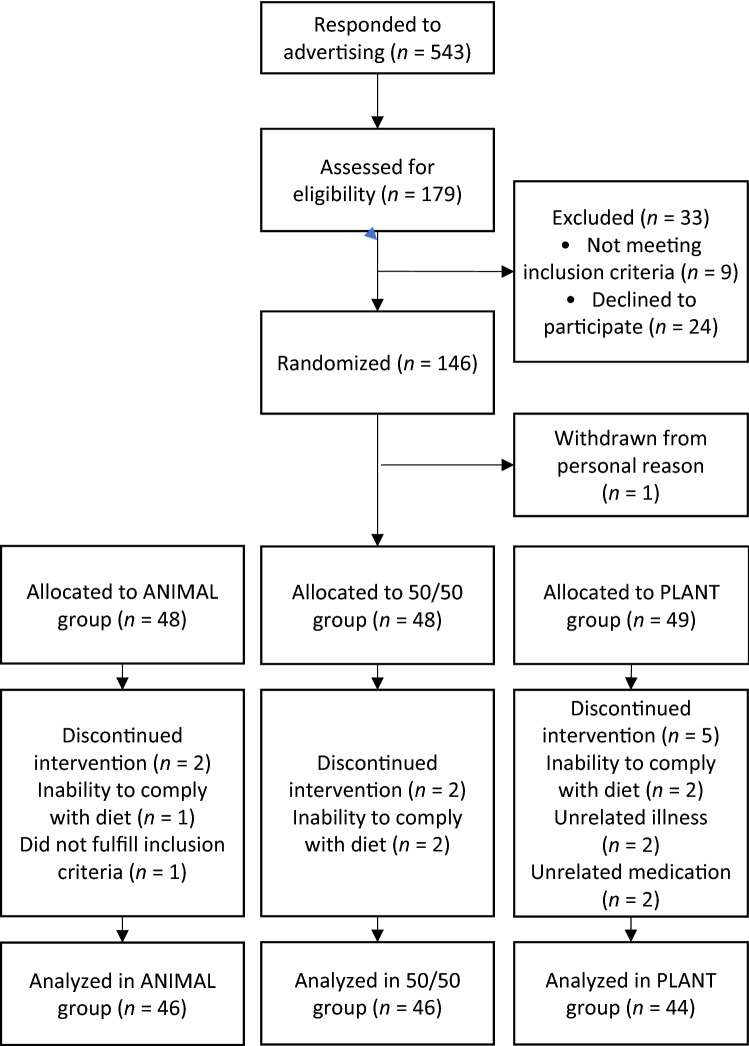
Flow chart of participants. ANIMAL, a diet containing 70% animal and 30% plant source proteins; 50/50, a diet containing equal proportions (50:50) of animal and plant-based protein sources; PLANT, a diet containing 30% animal and 70% plant proteins

The ScenoProt intervention study, conducted at the University of Helsinki, included 20- to 69-year-old healthy adult participants. Their body mass index (BMI) ranged between 18.5 and 35.0 kg/m^2^, and they volunteered to follow any of the three diets for the 12-week controlled trial in a parallel design. We recruited participants through newspaper advertisements, social media, and the university´s mailing lists. Exclusion criteria were fasting plasma glucose > 6.9 mmol/l and total cholesterol > 6.5 mmol/l, use of medication for diabetes or hypercholesterolemia, disorders of the intestinal or endocrine systems or of lipid metabolism, renal, or liver diseases, eating disorder, any malignant illness within the past five years, recent use of antibiotics (during the last three months), food allergies, extreme sports, smoking, and current pregnancy or lactation. Participant enrolment is shown in Fig. 1.

The screening was implemented from December 2016 to the beginning of March 2017, and the intervention periods began between January and March 2017. Altogether, 146 participants passed the screening and were stratified by sex and age and randomly allocated (allocation ratio 1:1:1) by the study PI to one of the three diet groups: ANIMAL, an animal-source protein diet representing an average Finnish diet and containing 70% animal- and 30% plant-source protein; 50/50, containing equal amounts of animal- and plant-source proteins; and PLANT, a plant-source protein diet containing 30% animal- and 70% plant-source protein. Participants were unaware of their diet group until they had completed baseline measures, after which their diet group was introduced using a colour code for each diet. The colour codes were used throughout the study. Participants were advised to discontinue use of dietary supplements and herbal or other natural remedies 2 weeks prior to the intervention period. Power calculation and sample size estimation of this research has been described in detail elsewhere [23, 24]. No power calculations regarding vitamin or mineral intakes or their biomarkers presented in this paper were carried out.

### Intervention diets

The intervention diets and compliance have been described in detail elsewhere (23, 24). In short, the three diets were designed to contribute, on average, 17% of energy from protein. The amount of red meat, poultry, and dairy products differed among the diet groups, being highest in the ANIMAL group, whereas the amounts of eggs and fish were the same in each diet group. In the 50/50 and PLANT diets, red meat, poultry and dairy products were partially replaced with plant protein sources such as cereal products, vegetable dishes including peas, lentils, chickpeas, tofu, faba beans, nuts, almonds, seeds, and plant-based dairy substitutes (Table 1). The food items supplied by the study provided on average 80% of the daily energy intake in all diet groups [23]. Participants were allowed to consume fruits, berries, dietary fats and oils, confectionery products, and other than plant-based dairy or vegetable substitute beverages as they usually did; they acquired these foods themselves.

**Table 1:** Consumption frequencies and daily consumption of specific foods and food groups in the intervention diets based on the delivered food items and diet instructions

	ANIMAL	50/50	PLANT
Sources of animal proteins			
Main dishes containing minced meat (times/wk)	2–3	1–2	0–1
Main dishes containing whole meat (times/wk)	2–3	1–2	0–1
Main dishes containing sausage (times/wk)	1	0–1	0–1
Sausages and cold cuts, including processed poultry (g/d)	35	23	11
Pork and beef (g/d)	64	43	21
Red and processed meat total (g/d)	99	65	32
Fish dishes (times/wk)	2	2	2
Fish (g/d)	36	36	36
Main dishes containing poultry (times/wk)	2–3	1–2	1
Poultry (g/d)	43	29	14
Eggs/wk (in dishes and pastries; boiled or fried)	4	4	4
Eggs (g/d)	31	31	31
Dairy products other than cheese (g/d)	400	250	125
Cheese (g/d)	40	25	10–15
Sources of plant proteins			
Main dishes based on peas, lentils, chickpeas, tofu, crushed soya beans, or faba beans as main ingredients (times/wk)	0–1	3–5	5–7
Vegetable patties, pizza, mushroom dishes (portions/wk)	1	2–3	2–3
Nuts, almonds, and seeds (g/d)	Occasionally	16	34
Plant-based dairy substitutes (other than cheese), g/d	0	150	250
Bread (rye and oat/wheat bread; slices of bread/d)	4–5	6	7
Bread (g/d)	120–150	180	210
Porridge and muesli (g/d, dry weight)	40	40–60	40–80
Whole-grain rice, pasta, couscous, quinoa (g/d, dry weight)	70	70–105	70–140
Potatoes (g/d, cooked)	120	0–120	0–120

Average daily consumption is presented as g/d. ANIMAL, a diet containing 70% animal and 30% plant source proteins; 50/50, a diet containing equal proportions (50:50) of animal and plant-based protein sources; PLANT, a diet containing 30% animal and 70% plant proteins

The participants visited the research unit weekly, when they were provided with most of their protein sources by the study to be consumed at home: meat, poultry, ready-made plant-protein products, bread, nuts and seeds, pulses (such as pea flower and dried pea groats), and fish. None of the ready-made plant-based foods or plant-based dairy substitutes supplied in this intervention were fortified with vitamin B-12, folic acid, iodine, iron, or zinc. The breads supplied contained iodized salt. The participants were instructed on how to implement their diet at a food level, and they received recipes for cooking.

### Assessment of baseline characteristics

The background data on age, sex, education, and previous use of dietary supplements (the type of the supplement but not the dose) were collected by a questionnaire. Multivitamins were assumed to include folic acid, vitamin B-12, iron, zinc, and iodine. In additional analysis, the data were dichotomized to previous users and non-users of vitamin B-12, folate, iodine, iron, and zinc supplements. BMI was calculated as weight (kg)/height (m^2^).

### Assessment of dietary intake

Four-day food records, including three weekdays and one weekend day, were collected prior to the start of the intervention and during the last week of the intervention. The participants were instructed to record portion sizes using weighing, package labels, or household measures. The baseline food records from two participants were not available. Food records were reviewed by nutritionists, and missing information was requested if needed. Vitamin and mineral intakes were calculated by the AivoDiet software (version 2.2.0.1, Aivo Oy, Turku, Finland), including the Fineli® Food Composition Database Release 16 (2013), maintained by the Finnish Institute for Health and Welfare [25]. Vitamin and mineral intakes at baseline and end point were calculated as means of daily intake. Each food item or mixed dish in the dataset was assigned to a food group, and retention factors (EuroFIR) [26] of folate, vitamin B-12, vitamin C, iron, and zinc were applied with a single factor per nutrient per food group. Total, animal-derived, plant-derived, and other sources of iron intake were calculated separately. All composite dishes and single food items were classified into 14 food categories based on a modified classification of the Fineli® database, and the proportions of the sources for nutrient intakes were calculated for each category. For analysing animal- and plant-derived iron intakes and the share of iodized salt of the total iodine intake, the data were disaggregated into ingredients of composite dishes and single food items and these were classified to 17 different ingredient categories, also based on a modified classification of the Fineli® database.

### Assessment of nutrient biomarkers

Blood samples were collected after overnight fast (10–12 h) at screening, baseline, and end point visits, and stored at − 70 °C until analysis. Plasma ferritin and transferrin receptor (TfR) concentrations, serum folate and holotranscobalamin II (holoTC), haemoglobin and high-sensitive C-reactive protein (hs-CRP) were analysed at Helsinki University Hospital Laboratories, Helsinki, Finland. All samples were analysed according to accredited standard methods. All biomarkers used were considered to most reliably represent the status of each nutrient [27–31]. In this study, no biomarker for zinc was examined because of the lack of sensitive clinical criteria to evaluate marginal or low zinc status [32].

Serum folate and holoTC were measured by immunochemiluminometric methods and plasma ferritin by photometric methods using the Abbott Architect iSR2000. The intra-assay coefficient of variation percentage (CV%) for folate was 8%, for holoTC 7%, and for ferritin 5%. Inter-assay CV% for folate was < 10% within the range 6–18 nmol/l, for holoTC < 7% within the range of 15–50 pmol/l, and for ferritin < 5% within the range of 20–323 µg/l. Plasma TfR was measured by immunoturbidimetric assay performed on the Abbott Architect c16000. For TfR, intra-assay CV% was 5.8% and inter-assay CV% < 8% within the range of 0.5–1.1 mg/l. Blood haemoglobin was analysed using photometric methods with inter-assay CV% ≤ 1.0% within the whole measurement range.

The participants collected 24-h urine samples at the beginning and at the end point of the intervention. 24-h urine iodine excretion (U-I) at the end point of the study was calculated based on urinary volume and urinary iodide concentration analysed at Vita laboratories in Helsinki by inductively coupled plasma - mass spectrometry with the Agilent 7700 ICP-MS. The CV% was 1.7% for intra-assay and 8.1% for inter-assay variation.

### Statistical analysis

Data are presented as means ± SD. Daily intakes are expressed also as energy-adjusted (µg or mg/MJ). Education was dichotomized to upper secondary or less and Bachelor’s degree or higher. For the analysis regarding folate and iron intake, the female participants were dichotomized according to Finnish mean age of menopause [33]: to ˂ 51 years (reproductive age) and ≥ 51 years (peri- and postmenopausal age). Differences among the diet groups in the intakes of vitamins and minerals at the end point were compared by one-way analysis of variance (ANOVA). Energy intake (MJ) was tested as a covariate for plant and animal-derived iron, but no change in the significance of the results was observed. The differences between nutrient biomarkers among dietary supplement users and non-users were tested by independent samples *t* tests. We contrasted nutrient biomarker concentration among the diet groups by analysis of covariance (ANCOVA), with adjustments made with the covariate: baseline concentrations*,* followed by post hoc comparisons, adjusted by the Bonferroni method. In addition, BMI, energy intake, age, sex, history of dietary supplement use for all biomarkers, and ﻿hs-CRP as indicator of iron status were verified as covariates, but no change in the significance of the results was observed. Multivariate significance test for indicators of iron status was done by using the multivariate linear model, where baseline values were used as covariates. Folate status was analysed separately for folic acid supplement users and non-users. We analysed biomarkers of iron status separately for male and female participants. The baseline value of TfR was missing for one subject in the 50/50 group and another subject in the PLANT group, as was the end point value of ferritin for one subject in the ANIMAL group. As an exception to other nutrient biomarker analyses, U–I was analysed by one-way ANOVA because baseline data were unavailable. The associations between dietary intake and nutrient biomarkers were determined with Spearman correlation.

*P* values < 0.05 were considered significant. Data analyses were done with R software (version 3.5.1 and Studio) (R Core Development Team) and with SPSS version 24 (25) (IBM).

## Results

### Baseline characteristics

Altogether, 136 of 145 randomized participants completed the study (Fig. 1), 79% of whom were females. Over half (52%) of the female participants were of reproductive age (< 51 years). Participants’ mean age was 48 (range 20–69) years, and mean BMI was 24.7 kg/m^2^. Most of the participants (69%) had an education higher than Bachelor’s degree (Table 2). Based on the background questionnaire, participants had moderate-intensity physical activity on average three times per week [23].

**Table 2:** Baseline characteristics of healthy adults (*n* = 136) who consumed intervention diets differing in animal and plant protein levels for 12 weeks

	ANIMAL (*n* = 46)	50%/50 (*n* = 46)	PLANT (*n* = 44)	All (*n* = 136)
Age (years)^a^	47.6 ± 14.5	47.2 ± 14.7	48.7 ± 14.0	47.8 ± 14.3
Sex				
Female (number/total)	37 (80)	36 (78)	34 (77)	107 (79)
Female of reproductive age (< 51 years)	19 (51)	20 (56)	17 (50)	56 (52)
Male	9 (20)	10 (22)	10 (23)	29 (21)
Education^b^				
Upper secondary or less	10 (22)	13 (28)	7 (16)	30 (22)
Bachelor’s degree or higher	33 (71)	26 (57)	35 (80)	94 (69)

Values are *n* (%). ANIMAL, a diet containing 70% animal and 30% plant source proteins; 50/50, a diet containing equal proportions (50:50) of animal and plant-based protein sources; PLANT, a diet containing 30% animal and 70% plant proteins

^a^Values are means ± SDs

^b^For education data, total *n* = 124 (data missing for 12 subjects)

### Dietary intake at end point

We aimed at the same proportion of energy from protein in all diet groups throughout the intervention, but at the end of the study the PLANT group had a slightly lower protein intake (15.2 E%) than the 50/50 (16.9 E%) and ANIMAL (18.2 E%) groups (ANIMAL vs. PLANT *P* ˂ 0.001 and 50/50 vs. PLANT *P* = 0.002). Energy, carbohydrate and total fat intake did not differ between the groups at the end of the intervention (*P* > 0.05), but fibre intake in the PLANT (*p* < 0.001) and in the 50/50 groups (*p* = 0.012) was higher than that in the ANIMAL group [23] (Supporting Information﻿ Table S1–S2). Supporting Information Table S1 shows the vitamin and mineral intakes at baseline. The proportion of dietary animal-source proteins among the study participants ranged from 61 to 65% and that of plant-source proteins from 35 to 39% of total protein intake among diet groups (Supporting InformationTable S1). Approximately, one-fourth of the participants had a history of folic acid or vitamin B-12 supplement use. Iodine supplement use was not common (Table 2).

**Fig. 2:**
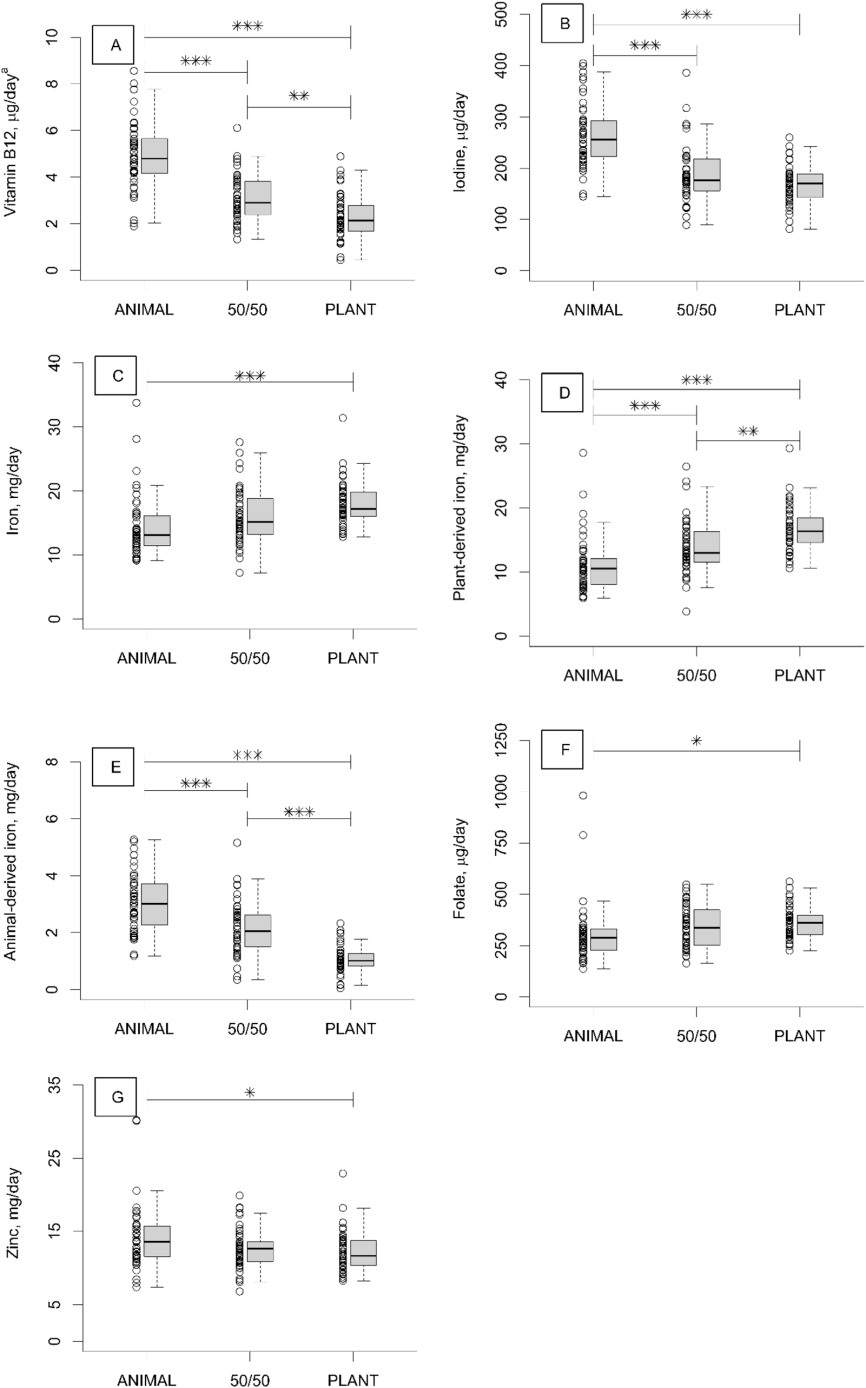
Intakes of (**A**) vitamin B-12, (**B**) iodine (one iodine intake (732.8 µg) in the PLANT diet is not shown in the fig.), (**C**) iron mg, (**D**) plant-derived iron, (**E**) animal-derived iron, (**F**) folate, and (**G**) zinc of healthy adults (*n* = 136) who consumed intervention diets differing in animal and plant protein levels for 12 weeks. ANIMAL, a diet containing 70% animal and 30% plant proteins (*n* = 46); 50/50, a diet containing equal proportions (50:50) of animal and plant proteins (*n* = 46), PLANT, a diet containing 30% animal and 70% plant proteins (*n* = 44). *Differences between the diet groups analysed by ANOVA with Bonferroni correction: *P < 0.05, ** *P* < 0.01, ***P < 0.001. ^a^One observed vitamin B-12 intake (16.6 µg) in the 50/50 group is not shown in the fig

At the end point, vitamin B-12 intakes were lower in the 50/50 (mean 3.4 µg/d) and PLANT (2.3 µg/d) groups than in the ANIMAL (4.9 µg/d) group (*P* < 0.001 for all), and lower in the PLANT group than in the 50/50 group (*P* = 0.007). Iodine intake was lower in the 50/50 (188.1 µg/d) and PLANT (180.9 µg/d) groups than in the ANIMAL (263.9 µg/d) group (*P* < 0.001 for all) (Fig. 2A–B, Supporting InformationTable S2). Folate intake was higher in the PLANT (364.1 µg/d) than in the ANIMAL (305.6 µg/d) group (*P* = 0.045), whereas zinc intake was lower in the PLANT (12.3 mg/d) than in the ANIMAL (14.1 mg/d) group (*P* = 0.044) (Fig. 2F-G, Supporting Information Table S2). No differences emerged in vitamin C intake among the diet groups (ANIMAL 160.4 mg/d, 50/50 147.4 mg/d, and PLANT 146.7 mg/d, *P* < 0.05) (Supporting Information Table S2).

Total iron intake was higher in the PLANT (mean 17.9 mg/d) than in the ANIMAL (14.3 mg/d) group (*P* < 0.001). Plant-derived iron intake was higher in the PLANT (16.7 mg/d) group than in the 50/50 (13.9 mg/d) (*P* = 0.004) and ANIMAL (11.2 mg/d) (*P* < 0.001) groups and higher in the 50/50 group than in the ANIMAL group (*P* = 0.005). Animal-derived iron intake was lower in the PLANT (1.0 mg/d) group than in the 50/50 (2.1 mg/d) and ANIMAL (3.0 mg/d) groups and lower in the 50/50 group than in the ANIMAL group (*P* < 0.001 for all). (Fig. 2C–E, Supporting Information Table S2). The proportion of animal-derived iron of total iron intake was 21% in the ANIMAL, 13% in the 50/50, and 6% in the PLANT group. The ratio of plant-derived to animal-derived iron (mg/mg) differed among all groups (*P* = 0.002 for all). The mean ratio of plant to animal iron was almost sevenfold when comparing the PLANT (28.8) with the ANIMAL group (4.0) (Supporting Information Table S2).

**Fig. 3:**
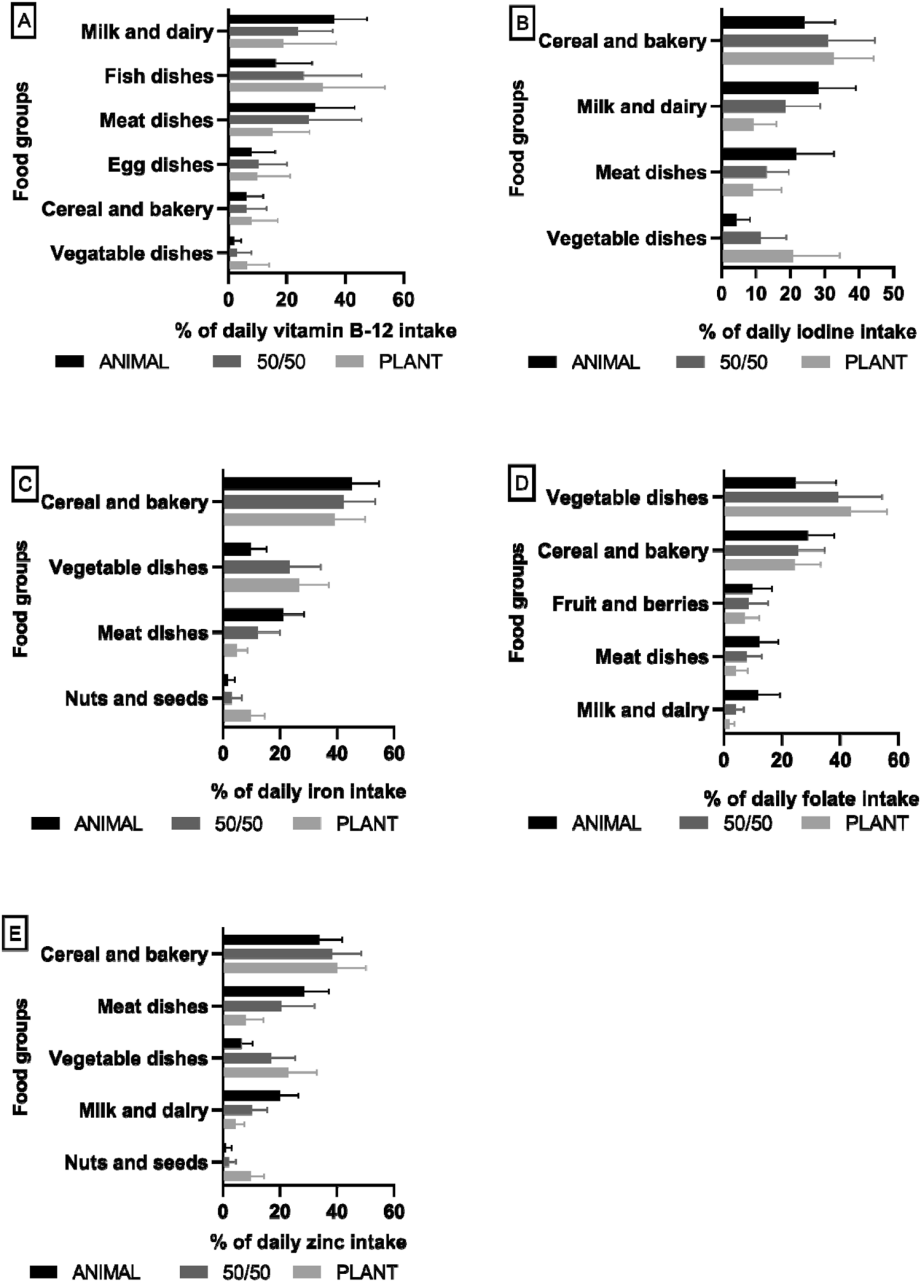
Main sources in descending order of (**A**) vitamin B-12, (**B**) iodine, (**C**) iron, (**D**) folate, and (**E**) zinc in the intervention diets presented as average proportions among healthy adults (*n* = 136) who consumed intervention diets differing in animal and plant protein levels for 12 weeks at the end point of the intervention. Line colours: black = ANIMAL, a diet containing 70% animal and 30% plant proteins (*n* = 46), dark grey = 50:50, a diet containing equal proportions (50:50) of animal and plant proteins (*n* = 46), grey = PLANT, a diet containing 30% animal and 70% plant proteins *(n* = 44)

The importance of vegetables and vegetable dishes as sources of all vitamins and minerals was pronounced in the diets containing more plant protein. In addition, in more plant protein-based groups, cereal and bakery products were important sources of zinc and iodine, while nuts and seeds served as important sources of iron and zinc (Fig. 3). The contribution of all food categories as sources of vitamins and minerals are described in Tables S3–S4**.** The analysis of ingredient categories as sources of iodine showed that the proportion of iodine intake from iodized salt was 31% in the ANIMAL group, 38% in the 50/50, and 39% in the PLANT group.

### Biomarkers of nutritional status and their correlations between vitamin and mineral intakes at end point

When analysing all diet groups together, the absolute intake (µg/day or mg/day) and the status of folate, vitamin B-12, and iodine correlated (folate, *r* = 0.221, *P* = 0.010; vitamin B-12, *r* = 0.301, *P* < 0.001; iodine, *r* = 0.495, *P* < 0.001) at the end point of the intervention.

**Fig. 4:**
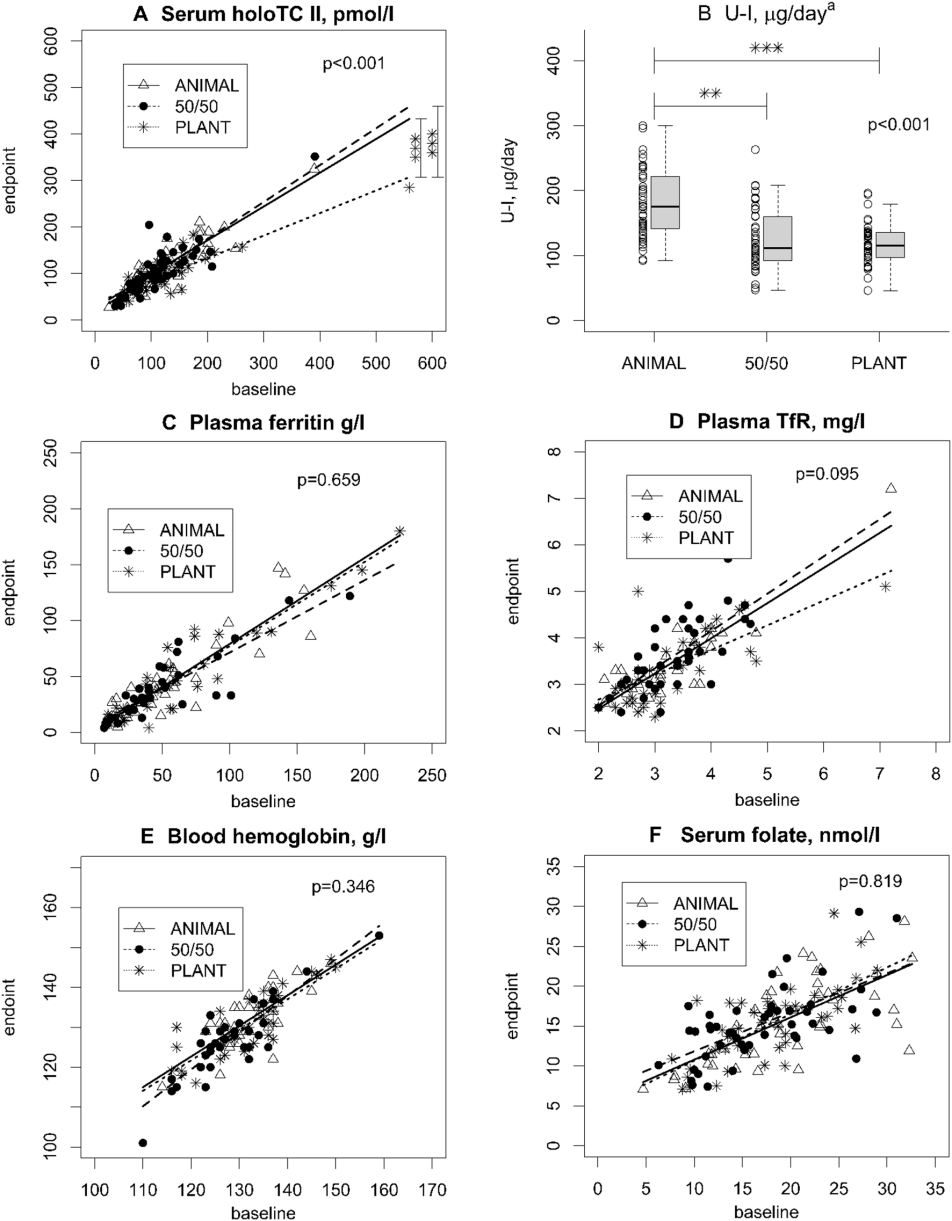
Plasma, serum, whole blood, and urinary biomarkers. (**A**) serum holoTC, (**B**) U-I, (**C**) plasma ferritin, (**D**) plasma TfR, (**E**) blood haemoglobin, and (**F**) serum folate of healthy adults (*n* = 136) who consumed intervention diets differing in animal and plant protein levels for 12 weeks. ANIMAL, a diet containing 70% animal and 30% plant proteins (*n* = 46); 50/50, a diet containing equal proportions (50:50) of animal and plant proteins (*n* = 46); PLANT, a diet containing 30% animal and 70% plant proteins (*n* = 44). *A–﻿B–**﻿B**: Differences between the diet groups analysed by ANCOVA adjusted for baseline value with Bonferroni correction﻿ (**A**) or ANOVA with Bonferroni correction (**B**): * *P* < 0.05, ** *P* < 0.01, *** *P* < 0.001. **C**–**E** include female participants only. Fig. C: ANIMAL *n* = 36, 50/50 *n* = 36, and PLANT *n* = 34; **D**–**E**: ANIMAL *n* = 37, 50/50 *n* = 36, and PLANT *n* = 34. ^a^Two concentrations: 1006 µg/day from the ANIMAL diet and 618 µg/day from the PLANT diet

Vitamin B-12 and iodine status differed among the intervention groups at the end point (*P* < 0.001 for both) (Fig. 4A–B, Supporting Information Table S5). HoloTC was lower in the PLANT group (mean 97.7 pmol/l) than in the other groups (105.8 pmol/l in the 50:50 and 122.1 pmol/l in the ANIMAL group) at the end point of the intervention (*P* < 0.001 for both) (Fig. 4A, Supporting Information Table S5). One participant (2.2%) in the ANIMAL group, five participants (10.9%) in the 50:50 group and four participants (9.1%) in the PLANT group had holoTC concentration under 50 pmol/l (Supporting information Fig. 1). U-I was also lower in the PLANT (128.7 µg/d, *P* = 0.002) and 50/50 (123.5 µg/d, *P*  < 0.001) groups than in the ANIMAL (197.4 µg/d) group (Fig. 4B, Supporting Information Table S5).

Three indicators of iron status were used, namely plasma ferritin, plasma TfR, and haemoglobin. We found no significant differences among the diet groups in indicators of iron status apart from the analyses stratified by sex (Fig. 4C–E, Supporting Information Table S5). Furthermore, the multivariate analysis combining all iron status biomarkers indicated no differences among the diet groups when adjusted by baseline concentrations (Pillai's Trace 0.129, *P* = 0.149, Supporting Information Table S6**)**.

We found no significant differences in folate status among the diet groups at the end point (Fig. 4F, Supporting Information Table S5). Serum folate was higher among participants who had previously used folic acid supplements than among their peers without a history of supplement use at baseline (22.6 vs. 16.7 nmol/l, *P* < 0.001) and end point (17.4 vs. 14.9 nmol/l, *P* = 0.015), but no significant differences emerged among diet groups in subgroup analyses stratified by supplement use (*P* > 0.05). Prior use of other dietary supplements did not contribute to higher concentrations of the other biomarkers (*P* > 0.05, data not shown).

## Discussion

In this 12-week randomized controlled trial among healthy adults, we studied the effects of partial replacement of animal-source proteins with plant-source proteins on intakes of critical vitamins and minerals and their status biomarkers using a whole-diet approach. A shift from mostly animal-sourced proteins towards more plant-sourced proteins led to significantly lower intakes and status of vitamin B-12 and iodine. However, despite the higher iron and folate intakes in the PLANT group than in the ANIMAL group, no differences in the indicators of iron or folate status among the diet groups were observed.

None of our participants were allowed to use dietary supplements, and the 50/50 and PLANT groups were supplied with non-fortified plant protein products. This allowed us to evaluate whether the small amount of animal-source foods in these diets was sufficient to secure adequate vitamin B-12 intake and status. We observed a significant dose-dependent decrease in B-12 intakes in the 50/50 and PLANT groups when partly replacing animal-source proteins with plant-source proteins. The lower vitamin B-12 intakes were reflected as lower serum holoTC concentrations, particularly in the PLANT group, where holoTC decreased 23% during the 12-week trial. We used holoTC as a vitamin B-12 status marker since it represents the active B-12 bound to transcobalamin II delivering B-12 to all tissues to be used for metabolic pathways [29]. It is considered to be the most sensitive marker for recent vitamin B-12 intake and also for vitamin B-12 deficiency [29, 34].

Our results are in line with those of Lerder et al*.* [14] who observed a significant decrease in dietary intake of vitamin B-12 and a decrease in both serum holoTC and B-12 concentrations in volunteers on an unsupplemented vegan diet after only a 4-week trial [14]. Cross-sectional evidence also shows that even partial restriction of animal-derived foods affects B-12 intake. In the large EPIC-Oxford cohort, omnivores had the highest B-12 intake, followed by progressively lower intakes by fish-eaters, lacto-ovo vegetarians, and vegans [12]. Interestingly, high consumption of dairy products and especially milk has been associated with better vitamin B-12 status, which might indicate better bioavailability of vitamin B-12 from dairy products than from meat and eggs [35, 36]. Thus, cutting considerably the consumption of milk and dairy products in the PLANT group could at least partly explain the rather sharp decrease in vitamin B-12 status. Even though the mean intake of vitamin B-12 in the PLANT group was only half of that in the ANIMAL group, it remained above the recommended intake of 2 µg/d given in the Nordic Nutrition Recommendations (NNR) [37]. It is noteworthy that B-12 recommendations vary worldwide [29]. For example, the European Food Safety Authority has set 4 μg/d as an adequate intake for B-12, based on reported intakes of B-12 for maintaining reference ranges of several status markers of serum B-12 in healthy adults [38]. During our trial, holoTC values of three participants belonging to the 50/50 and PLANT groups dropped below 35 pmol/l, which is considered the criterion for vitamin B-12 deficiency**.** Our results support the suggestion made by the NNR on considering vitamin B12 supplementation if following a vegetarian diet [37]. Taken together, our results strongly challenge the long-held notion that when a well-nourished person changes from an omnivorous diet to a flexitarian diet, i.e. a plant-based diet containing limited amounts of animal-source foods, vitamin B-12 status will not be compromised.

Although average iodine intake and status reached the recommendations in all diet groups [37, 39], we observed more deficiency and a significantly lower iodine intake and status in the 50/50 and PLANT groups than in the ANIMAL group. As the majority of the subjects were female, it is worth noting that the average intakes in the 50/50 and PLANT groups did not meet the suggested demands during pregnancy and lactation [39]. Our finding is consistent with previous studies indicating lower iodine intake and status in vegetarians and vegans [8, 13, 15]. In the current Finnish diet, similarly to the ANIMAL group in the present study, about one-third of iodine is obtained from dairy products [40]. In Finland, the nutritional policy measures have gradually improved the iodine status of the population, as the use of iodized salt in the food and bakery industry and food services has become common [40, 41]. However, in some European countries, mild-to-moderate iodine deficiency is still common in the adult population and in pregnant females [42]. When moving toward more sustainable diets with increased replacement of animal-based iodine sources with plant-based alternatives, emphasis should be placed on systematically using iodized salt in salt-containing industrial food products and fortifying plant-based dairy substitutes with iodine. If necessary, countries should also consider other policy strategies, such as recommending iodine supplementation to all pregnant women—a strategy not currently in place in Finland. In our study, iodine intake was most likely overestimated due to the absence of retention factors since possible effects of preservation and cooking losses, ranging from 6 to 52%, were not taken into account [43].

In our study, the PLANT group had the highest iron intakes. Previous cross-sectional studies have also consistently reported that especially vegans but also vegetarians have higher iron intakes than omnivores [8, 10, 16, 17]. Regardless of their higher iron intakes, a recent meta-analysis suggested that vegetarians are more likely to have lower serum ferritin concentrations than non-vegetarians [44], indicating a markedly lower bioavailability of iron from plant-based diets than from omnivorous diets. Indeed, it has been shown, using an extrinsic ^59^Fe isotope, that non-heme iron absorption from the lacto-ovovegetarian diet was 70% less than from the non-vegetarian diet in adult females [19]. We used three established biomarkers for iron status, namely plasma ferritin, plasma TfR, and haemoglobin [28], but found no differences in any of them among the diet groups at the end point of the 12-week intervention. It is possible that the duration of the study was not sufficiently long to observe changes in the indicators of iron status. This could be due to adaptation of participants to dietary iron bioavailability by increasing non-heme iron absorption to maintain iron homeostasis [18]. Alternatively, the enhancing and inhibiting factors affecting iron bioavailability in the diets containing more plant protein may have outbalanced each other. Animal-derived iron intake decreased, and thus, presumably, also better available heme iron intake decreased, while the intake of non-heme iron increased substantially. The bioavailability of heme iron seems to be constant regardless of dietary regime [28], but the absorption of non-heme iron is heavily affected by inhibitors, such as phytates and polyphenols, commonly present in legumes, soy, and whole grains and, on the other hand, enhancers such as vitamin C and muscle tissue from animal foods [28, 45]. It is noteworthy that the mean intake of vitamin C in all diet groups was about twofold higher than the recommended intake [37], and it may have considerably enhanced non-heme iron bioavailability in the groups containing more plant proteins. In addition, even the PLANT group consumed some animal-derived iron, along with muscle tissue, which may have enhanced non-heme iron bioavailability. All of these enhancing factors together could, at least to some extent, overcome the inhibitory factors in more plant protein-containing diets.

In our study, zinc intake was lowest in the PLANT group, which is in accordance with the previous studies concerning plant-based diets [8, 10, 12]. In all diet groups, zinc intakes were above the RI (7 mg/d for females and 9 mg/d for males) [37] both at the baseline and at the end point. However, it should be taken into account that, based on the IOM report, the requirement for dietary zinc may be as much as 50 percent higher particularly for strict vegetarians whose major food staples are grains and legumes [22]. The main sources of zinc in more plant protein containing diets, i.e. whole grains, legumes, nuts, and seeds, are also high in phytic acid, which is a main inhibitor of zinc bioavailability. In this study, we did not examine nutritional zinc status because more research is still needed for a specific, sensitive, and field-friendly zinc biomarker to evaluate the zinc status of individuals or populations [32].

Higher folate intake in the diet containing 70% plant-source proteins was in line with earlier studies on plant-based diets [8–10]. Despite the increase in intake, no differences were observed in folate status between the diets. In each intervention group, folate intake both at baseline and at end point was higher than in general population in Finland [40]. The previous folic acid supplementation users, most of whom were in the PLANT group, seemed to have higher folate status at the baseline and end point. Since the half-life of folic acid is about 100 days [46], the previous use of supplementation may have hidden the effects of the intervention diets on the folate status. Thus, longer study period would have needed to observe changes in folate status. In addition, serum folate is highly responsive to folic acid and might give a poorer response to natural food folates [30].

Notable strengths of our study are the relatively long duration of the trial, excellent compliance of the study participants, and a comprehensive assessment of dietary intakes, including separate analyses of animal- and plant-derived iron. The participants were not allowed to consume dietary supplements, and the foods and drinks provided to them were not fortified with the assessed nutrients, with the exception of iodized salt as an ingredient in bakery and industrial products. This allowed us to better evaluate the effects of the diets on vitamin and mineral intake and status. Our female-dominated sample was highly educated, and based on high intakes of fibre [23] and folate at baseline, probably more health conscious than Finns on average, which may have impacted the effects of the intervention diets and resulted in unbalanced subgroup analyses by sex.

To conclude, we demonstrated in the 12-week trial with a whole-diet approach that vitamin B-12 and iodine are among the most critical nutrients when partly replacing animal-source protein with plant protein. The adequate intake of these nutrients needs to be ensured when recommending more plant-based diets on a population level. Our data call for longer trials to confirm the effects of more plant-based diets on iron status. It remains to be evaluated what type of fortification and supplementation strategies can secure vitamin B-12 and iodine intakes in different flexitarian diets with varying proportions of animal-source foods.

## Supplementary Information

Below is the link to the electronic supplementary material.

Supplementary file1 (DOCX 64 kb)

## Abbreviations

50/50: A diet containing equal amounts of animal and plant proteins
ANCOVA: Analysis of covariance
ANIMAL: A diet containing 70% animal and 30% plant protein
ANOVA: Analysis of variance
holoTC: Holotranscobalamin II
hs-CRP: High-sensitivity C-reactive protein
NNR: Nordic nutrition recommendations
PLANT: A diet containing 30% animal and 70% plant protein
TfR: Transferrin receptor
U-I: 24-Hour urinary iodine excretion

## Declarations

### Data availability

Data and analytical code described in the manuscript will be made available upon request pending application and approval in agreement with the EU General Data Protection Regulation.

### Code availability

Code book and analytic code of the data described in the manuscript will be made available upon request pending application and approval in agreement with the EU General Data Protection Regulation.
