# Supplementary material for: Re﻿placing dietary animal-source proteins with plant-source proteins changes dietary intake and status of vitamins and minerals in healthy adults: a 12-week randomized controlled trial
Source: Eur J Nutr. 2021 Nov 27;61(3):1391–404. doi: 10.1007/s00394-021-02729-3 (PMC8921037; doi:10.1007/s00394-021-02729-3)
Supplement: Supplementary file 1 — Supplementary file1 (DOCX 64 kb) [file 394_2021_2729_MOESM1_ESM.docx]

**Figure S1.** HoloTC consentrations of healthy adults (*n* = 136) who consumed intervention diets differing in animal-source and plant-source proteins for 12 weeks^†^.

**
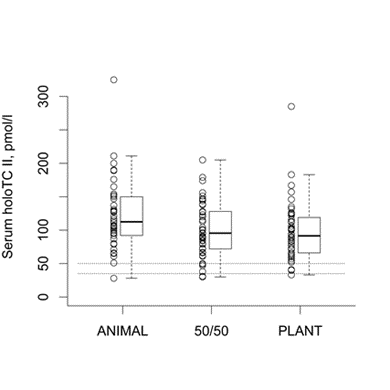
**

ANIMAL, a diet containing 70% animal and 30% plant proteins (n = 46); 50/50, a diet containing equal proportions (50:50) of animal and plant proteins (n = 46), PLANT, a diet containing 30% animal and 70% plant proteins (n = 44).

Dotted lines at 35 pmol/l and 50 pmol/l presents deficiency and marginal status

†One vitamin holoTC consentration (352 pmol/l) in the 50/50 group is not shown in the figure.

**Table S1** Baseline (0 wk) nutrient intakes of healthy adults (*n* = 136) who consumed intervention diets differing in animal and plant protein levels for 12 weeks^a^

|  | ANIMAL  (*n* = 46)^c^ | 50/50  (*n* = 46) | PLANT  (*n* = 44)^d^ | All  (*n* = 136) |
| --- | --- | --- | --- | --- |
| Energy, kJ | 9124 ± 2179 | 8732 ± 1959 | 8788 ± 1675 | 8881 ± 1946 |
| Dietary animal/plant protein, % | 65/35 | 61/39 | 62/38 | 62/38 |
| Protein, E% | 18.5 ± 3.2 | 17.3 ± 3.1 | 17.7 ± 3.4 | 17.8 ± 3.3 |
| Carbohydrates, E% | 39.0 ± 6.6 | 40.3 ± 5.8 | 40.7 ± 5.7 | 40.0 ± 6.1 |
| Fat, E% | 37.7 ± 5.7 | 37.2 ± 6.1 | 36.6 ± 5.2 | 37.2 ± 5.7 |
| Vitamin B-12, µg | 6.7 ± 4.7 | 6.3 ± 3.3 | 7.9 ± 6.8 | 6.9 ± 5.1 |
| Vitamin B-12, µg/MJ | 0.7 ± 0.5 | 0.7 ± 0.4 | 0.9 ± 0.8 | 0.8 ± 0.6 |
| Iodine, µg | 275.8 ± 84.0 | 257.4 ± 115.8 | 255.0 ± 95.5 | 262.8 ± 99.2 |
| Iodine, µg/MJ | 30.9 ± 9.1 | 29.0 ± 8.6 | 29.4 ± 11.2 | 29.8 ± 9.7 |
| Iron, mg | 14.5 ± 4.7 | 14.1 ± 3.7 | 14.2 ± 3.7 | 14.3 ± 4.0 |
| Iron, mg/MJ | 1.6 ± 0.3 | 1.6 ± 0.3 | 1.6 ± 0.3 | 1.6 ± 0.3 |
| Iron, mg, female^e^ | 13.9 ± 4.5 | 13.2 ± 2.8 | 13.8 ± 3.8 | 13.6 ± 3.7 |
| Iron, mg, male^e^ | 17.3 ± 5.0 | 17.3 ± 4.7 | 15.8 ± 3.1 | 16.7 ± 4.2 |
| Plant-derived iron, mg^e^ | 11.0 ± 4.1 | 11.0 ± 3.0 | 10.9 ± 3.6 | 11.0 ± 3.6 |
| Animal-derived iron, mg^f^ | 3.5 ± 1.8 | 3.2 ± 1.7 | 3.3 ± 2.1 | 3.3 ± 1.8 |
| Ratio of plant- to animal-derived iron, mg/mg | 3.8 ± 2.3 | 4.5 ± 2.9 | 5.4 ± 5.4 | 4.6 ± 3.8 |
| Folate, µg | 308.7 ± 122.2 | 314.3 ± 113.1 | 316.7 ± 130.7 | 313.2 ± 121.1 |
| Folate, µg/MJ | 33.9 ± 10.8 | 36.3 ± 10.7 | 36.4 ± 14.7 | 35.5 ± 12.1 |
| Zinc, mg | 13.7 ± 4.5 | 12.3 ± 3.5 | 12.4 ± 3.0 | 12.8 ± 3.8 |
| Zinc, mg/MJ | 1.5 ± 0.3 | 1.4 ± 0.2 | 1.4 ± 0.2 | 1.4 ± 0.2 |
| Vitamin C, mg | 161.0 ± 75.8 | 161.2 ± 96.1 | 167.6 ± 74.0 | 163.2 ± 82.2 |
| Vitamin C, mg/MJ | 17.6 ± 7.5 | 18.5 ± 9.9 | 19.9 ± 10.3 | 18.6 ± 9.3 |
| Previous vitamin B-12 supplement users^b^ | 10 (22) | 8 (17) | 14 (32) | 32 (24) |
| Previous iodine supplement users^b^ | 6 (13) | 5 (11) | 5 (11) | 16 (12) |
| Previous iron supplement users^b^ | 5 (11) | 7 (15) | 8 (18) | 20 (15) |
| Previous folic acid supplement users^b^ | 9 (20) | 8 (17) | 14 (32) | 31 (23) |
| Previous zinc supplement users^b^ | 11 (24) | 8 (17) | 11 (25) | 30 (22) |
|  |  |  |  |  |

^2^Values are means ± SDs, ANIMAL, a diet containing 70% animal and 30% plant source proteins; 50/50, a diet containing equal proportions (50:50) of animal and plant-based protein sources; PLANT, a diet containing 30% animal and 70% plant proteins. Data were analyzed by ANOVA with Bonferroni correction.

^b^Values are *n*(%)

^c^For nutrient intakes, *n* = 45

^d^For nutrient intakes, *n* = 43

^e^for females ANIMAL n = 37, 50/50 n=36, and PLANT n = 33, for males ANIMAL n = 8, 50/50 n =10, and PLANT n = 10

^c^Plant-derived iron includes iron from vegetables, potatos, fruits, berries, cereals, sugar and confectionery, nuts and seeds, plant-based dairy substitutes, beverages and alcoholic beverages, and legumes.

^d^Animal-derived iron includes iron from milk, fish, crustaceans, molluscs, meat, and egg.

**Table S2** Daily energy intake, non-adjusted and adjusted (/MJ) nutrient intakes among healthy adults (*n* = 136) who consumed intervention diets differing in animal and plant protein levels for 12 weeks at the end point of the intervention^a^

|  | ANIMAL  (*n* = 46) | 50/50  (*n* = 46) | PLANT  (*n* = 44) | *P* |
| --- | --- | --- | --- | --- |
| Energy, kJ | 9221.5 ± 1947.8 | 8653.2 ± 1839.4 | 9096.1 ± 1434.1 | 0.201 |
| Dietary animal/plant protein, % | 68/32 | 52/48 | 26/74 |  |
| Protein, E% | 18.2 ± 3.1 | 16.9 ± 2.2 | 15.2 ± 2.0 | <0.001^†,‡^ |
| Carbohydrates, E% | 39.8 ± 5.7 | 41.0 ± 4.6 | 42.2 ± 5.2 | 0.108 |
| Fat, E% | 36.9 ± 4.9 | 37.1 ± 5.8 | 37.1 ± 5.1 | 0.974 |
| Vitamin B-12, µg | 4.9 ± 1.5 | 3.4 ± 2.2 | 2.3 ± 1.0 | <0.001^*,†,‡^ |
| Vitamin B-12, µg/MJ | 0.5 ± 0.1 | 0.4 ± 0.2 | 0.3 ± 0.1 | <0.001^*,†,‡^ |
| Iodine, µg | 262.9 ± 63.5 | 188.1 ± 56.4 | 180.9 ± 92.9 | <0.001^*,†^ |
| Iodine, µg/MJ | 28.8 ± 4.8 | 21.8 ± 4.6 | 19.9 ± 9.3 | <0.001^*,†^ |
| Iron, mg | 14.3 ± 4.9 | 16.0 ± 4.2 | 17.9 ± 3.6 | 0.001^†^ |
| Iron, mg, female^b^ | 13.6 ± 3.4 | 15.4 ± 3.5 | 17.0 ± 2.7 | <0.001^†^ |
| Iron, mg, male^b^ | 17.6 ± 8.3 | 18.4 ± 5.8 | 21.0 ± 4.6 | 0.473 |
| Iron, mg/MJ | 1.5 ± 0.3 | 1.9 ± 0.3 | 2.0 ± 0.2 | <0.001^*,†^ |
| Iron, mg/MJ, female^b^ | 1.5 ± 0.2 | 1.9 ± 0.3 | 2.0 ± 0.2 | <0.001^*,†^ |
| Iron, mg/MJ, male^b^ | 1.6 ± 0.4 | 1.8 ± 0.3 | 2.0 ± 0.3 | 0.054 |
| Plant-derived iron, mg^c^ | 11.2 ± 4.3 | 13.9 ± 4.2 | 16.7 ± 3.6 | <0.001^*,†,‡^ |
| Animal-derived iron, mg^d^ | 3.0 ± 1.0 | 2.1 ± 0.9 | 1.1 ± 0.5 | <0.001^*,†,‡^ |
| Ratio of plant to animal-derived iron, mg/mg | 4.0 ± 1.6 | 8.7 ± 8.3 | 28.8 ± 59.1 | 0.002^†,‡^ |
| Folate, µg | 305.6 ± 144.9 | 343.2 ± 103.5 | 364.1 ± 77.2 | 0.047^†^ |
| Folate, µg/MJ | 32.4 ± 9.2 | 39.7 ± 9.7 | 40.3 ± 8.2 | <0.001^*,†^ |
| Zinc, mg | 14.1 ± 4.5 | 12.7 ± 2.8 | 12.3 ± 2.8 | 0.036^†^ |
| Zinc, mg/MJ | 1.5 ± 0.2 | 1.5 ± 0.2 | 1.4 ± 0.2 | 0.001^†,‡^ |
| Vitamin C, mg | 160.4 ± 115.2 | 147.4 ± 65.0 | 146.7 ± 65.6 | 0.692 |
| Vitamin C, mg/MJ | 16.6 ± 8.5 | 16.9 ± 6.7 | 16.4 ± 7.3 | 0.951 |

^a^Values are means ± SDs, ANIMAL, a diet containing 70% animal and 30% plant source proteins; 50/50, a diet containing equal proportions (50:50) of animal and plant-based protein sources; PLANT, a diet containing 30% animal and 70% plant proteins. Data were analyzed by ANOVA with Bonferroni correction.

**^b^**for females ANIMAL *n* = 37, 50/50 *n*=36, and PLANT *n* = 34, for males ANIMAL *n* = 9, 50/50 *n* =10, and PLANT *n* = 10

^c^“Plant-derived iron” includes iron from vegetable, potatos, fruits, berries, cereals, sugar and confectionery, nuts and seeds, plant-based dairy substitutes, beverages and alcoholic beverages, and legumes.

^d“^Animal-derived iron” includes milk, fish, crustaceans, molluscs, meat, and egg.

*post hoc comparison (Bonferroni correction) between ANIMAL and 50/50, *P* < 0.05

^†^post hoc comparison (Bonferroni correction) between ANIMAL and PLANT, *P* < 0.05

^‡^post hoc comparison (Bonferroni correction) between 50/50 and PLANT, *P* < 0.05

**Table S3** Sources of vitamins (folate and vitamin B-12) among healthy adults (*n* = 136) who consumed intervention diets differing in animal and plant protein levels for 12 weeks at the end point of the intervention, presented as average proportions based on 4-day food record data. Three major sources of folate and vitamin B-12 are shown in bold^a^

| Food group | Folate | | | Vitamin B-12 | | |
| --- | --- | --- | --- | --- | --- | --- |
|  | ANIMAL | 50/50 | PLANT | ANIMAL | 50/50 | PLANT |
| Meat dishes | **12.2 ± 6.6** | 7.8 ± 5.1 | 4.1 ± 4.0 | **29.6 ± 13.6** | **27.5 ± 18.1** | **15.2 ± 12.6** |
| Milk and dairy products | 11.7 ± 7.5 | 4.1 ± 2.8 | 1.8 ± 1.8 | **36.1 ± 11.3** | **23.8 ± 11.9** | **18.7 ± 18.2** |
| Fish dishes | 3.5 ± 2.5 | 3.0 ± 2.6 | 2.7 ± 2.3 | **16.2 ± 12.5** | **25.9 ± 19.7** | **32.1 ± 21.4** |
| Egg dishes | 2.7 ± 2.7 | 1.9 ± 2.3 | 1.4 ± 2.0 | 7.8 ± 8.2 | 10.2 ± 9.9 | 9.7 ± 11.3 |
| Cereals and bakery products | **29.0 ± 9.0** | **25.5 ± 9.1** | **24.4 ± 8.9** | 6.0 ± 5.9 | 6.0 ± 7.1 | 7.8 ± 9.1 |
| Vegetables and vegetable dishes | **24.7 ± 13.8** | **39.2 ± 15.1** | **43.6 ± 12.4** | 2.0 ± 2.4 | 2.9 ± 4.9 | 6.4 ± 7.5 |
| Nuts and seeds | 0.8 ± 1.5 | 1.6 ± 2.5 | 4.8 ± 2.9 | 0 ± 0 | 0 ± 0 | 0 ± 0 |
| Plant-based dairy substitutes | 0 ± 0.2 | 2.8 ± 4.7 | 4.9 ± 9.7 | 0.1 ± 0.8 | 1.8 ± 4.7 | 6.4 ± 13.9 |
| Potatoes and potato dishes | 2.0 ± 2.5 | 1.2 ± 2.3 | 0.5 ± 0.9 | 0.2 ± 0.4 | 0.2 ± 0.7 | 0.1 ± 0.4 |
| Fruits, berries, and fruit and berry dishes | 9.7 ± 6.8 | **8.5 ± 6.8** | **7.2 ± 4.9** | 0.1 ± 0.4 | 0.5 ± 1.3 | 0.1 ± 0.4 |
| Beverages | 2.7 ± 2.7 | 3.0 ± 9.0 | 2.6 ± 3.7 | 0.9 ± 2.8 | 0.3 ± 1.6 | 1.7 ± 4.8 |
| Sugar and confectionery | 0.2 ± 0.3 | 0.3 ± 0.4 | 0.1 ± 0.2 | 0 ± 0.1 | 0 ± 0.2 | 0.1 ± 0.5 |
| Miscellaneous ^b^ | 0.5 ± 1.1 | 0.3 ± 0.7 | 1.2 ± 3.7 | 0.6 ± 2.3 | 0.4 ± 1.5 | 1.0 ± 3.1 |
| Fat spreads, oils, and dressings | 0.3 ± 0.5 | 0.8 ± 1.9 | 0.7 ± 1.3 | 0.4 ± 1.2 | 0.5 ± 1.2 | 0.7 ± 1.9 |

^a^Values are means ± SDs, ANIMAL, a diet containing 70% animal and 30% plant source proteins; 50/50, a diet containing equal proportions (50:50) of animal and plant-based protein sources; PLANT, a diet containing 30% animal and 70% plant proteins. Data were analyzed by ANOVA with Bonferroni correction.

^b^Food group “Miscellaneous” includes dried fruits and berries, snacks, spices, piquant sauces, weight loss products, meal replacements, protein powders, protein bars, and other miscellaneous foods.

**Table S4** Sources of minerals (iron, zinc and iodine) among healthy adults (*n* = 136) who consumed intervention diets differing in animal and plant protein levels for 12 weeks at the end point of the intervention, presented as average proportions (% ± SD) based on 4-day food record data. Three major sources of iron, zinc, and iodine are shown in bold^a^

| Food group | Iron |  |  | Zinc |  |  | Iodine |  |  |
| --- | --- | --- | --- | --- | --- | --- | --- | --- | --- |
|  | ANIMAL | 50/50 | PLANT | ANIMAL | 50/50 | PLANT | ANIMAL | 50/50 | PLANT |
| Meat dishes | **21.0 ± 7.4** | **12.2 ± 7.8** | 4.8 ± 3.9 | **28.3 ± 8.7** | **20.5 ± 11.6** | 8.0 ± 6.3 | **21.7 ± 11.0** | **13.1 ± 6.4** | 9.1 ± 8.3 |
| Milk and dairy products | 2.7 ± 1.9 | 1.1 ± 0.8 | 0.5 ± 0.5 | **19.8 ± 6.8** | 10.1 ± 5.5 | 4.5 ± 3.0 | **28.1 ± 10.9** | **18.6 ± 10.1** | 9.2 ± 6.8 |
| Fish dishes | 2.7 ± 2.2 | 2.3 ± 2.8 | 1.6 ± 1.3 | 2.5 ± 1.9 | 2.8 ± 3.0 | 2.7 ± 2.1 | 10.0 ± 7.2 | 11.0 ± 8.2 | **12.4 ± 9.9** |
| Egg dishes | 3.4 ± 3.7 | 2.6 ± 3.0 | 1.7 ± 2.2 | 2.0 ± 2.3 | 1.8 ± 2.0 | 1.4 ± 1.8 | 3.9 ± 5.0 | 4.2 ± 4.0 | 3.2 ± 3.9 |
| Cereals and bakery products | **45.0 ± 9.6** | **42.3 ± 10.9** | **39.0 ± 10.8** | **33.8 ± 7.9** | **38.2 ± 10.2** | **39.9 ± 10.2** | **24.1 ± 8.9** | **31.0 ± 13.5** | **32.5 ± 11.6** |
| Vegetables and vegetable dishes | **9.6 ± 5.8** | **23.3 ± 11.0** | **26.6 ± 10.4** | 6.5 ± 3.7 | **16.9 ± 8.4** | **22.9 ± 9.9** | 4.2 ± 4.1 | 11.2 ± 7.5 | **20.7 ± 13.7** |
| Nuts and seeds | 1.6 ± 2.6 | 3.1 ± 3.6 | **9.6 ± 5.0** | 1.0 ± 2.1 | 2.2 ± 2.4 | **9.8 ± 4.6** | 0.1 ± 0.2 | 0.2 ± 0.2 | 0.9 ± 0.7 |
| Plant-based dairy substitutes | 0.1 ± 0.3 | 2.5 ± 3.4 | 4.4 ± 6.0 | 0 ± 0.3 | 1.8 ± 2.7 | 3.2 ± 3.3 | 0 ± 0.1 | 0.7 ± 1.8 | 1.3 ± 2.3 |
| Potatoes and potato dishes | 1.8 ± 2.1 | 1.2 ± 2.2 | 0.5 ± 0.8 | 0.9 ± 1.1 | 0.7 ± 1.2 | 0.4 ± 0.6 | 1.8 ± 2.8 | 1.4 ± 2.6 | 0.7 ± 1.2 |
| Fruits, berries, and fruit and berry dishes | 5.0 ± 2.6 | 3.9 ± 2.2 | 4.2 ± 2.2 | 2.3 ± 2.9 | 2.0 ± 1.3 | 2.7 ± 2.4 | 1.2 ± 1.1 | 1.7 ± 1.3 | 1.7 ± 1.6 |
| Beverages | 3.1 ± 2.2 | 2.2 ± 2.3 | 2.8 ± 2.2 | 1.2 ± 0.8 | 0.9 ± 0.7 | 1.5 ± 2.1 | 2.2 ± 1.6 | 2.7 ± 3.7 | 3.8 ± 2.5 |
| Sugar and confectionery | 2.9 ± 3.7 | 2.1 ± 2.3 | 1.5 ± 2.5 | 1.2 ± 1.2 | 1.4 ± 1.8 | 0.9 ± 1.3 | 1.2 ± 1.6 | 1.8 ± 2.5 | 1.5 ± 2.6 |
| Miscellaneous^b^ | 0.8 ± 1.4 | 0.6 ± 1.2 | 2.1 ± 4.2 | 0.3 ± 0.7 | 0.3 ± 0.7 | 1.5 ± 4.2 | 0.6 ± 1.3 | 1.0 ± 1.5 | 1.8 ± 3.6 |
| Fat spreads, oils, and dressings | 0.3 ± 0.4 | 0.6 ± 1.1 | 0.7 ± 1.8 | 0.2 ± 0.2 | 0.4 ± 0.8 | 0.6 ± 1.8 | 0.9 ± 1.3 | 1.4 ± 1.8 | 1.2 ± 1.4 |

^a^Values are means ± SDs, ANIMAL, a diet containing 70% animal and 30% plant source proteins; 50/50, a diet containing equal proportions (50:50) of animal and plant-based protein sources; PLANT, a diet containing 30% animal and 70% plant proteins. Data were analyzed by ANOVA with Bonferroni correction.

^b^Food group “Miscellaneous” includes dried fruits and berries, snacks, spices, piquant sauces, weight loss products, meal replacements, protein powders, protein bars, and other miscellaneous foods.

**Table S5** Nutritional status among healthy adults (*n* = 136) who consumed intervention diets differing in animal and plant protein levels for 12 weeks at baseline (0 wk) and end point (12 wk)^a^

|  |  | ANIMAL (*n*=46) | 50/50  (*n*=46) | PLANT (*n*=44) | *P* |
| --- | --- | --- | --- | --- | --- |
| Body mass index, kg/m^2^ | 0 wk | 24.7 ± 4.1 | 24.4 ± 3.9 | 25.2 ± 3.7 |  |
|  | 12 wk | 25.0 ± 4.3 | 24.5 ± 4.1 | 25.2 ± 3.7 | 0.679 |
|  |  |  |  |  |  |
| Serum holoTC, pmol/l | 0 wk | 131.5 ± 62.6 | 112.7 ± 59.8 | 126.0 ± 81.5 |  |
|  | 12 wk | 122.1 ± 51.9 | 105.8 ± 54.7 | 97.7 ± 45.2 | <0.001^‡,ͳ^ |
|  |  |  |  |  |  |
| U–I, µg/day* | 0 wk | na | na | na |  |
|  | 12 wk | 197.4 ± 132.4 | 123.5 ± 47.2 | 128.7 ± 82.0 | <0.001^†,‡^ |
|  |  |  |  |  |  |
| Plasma ferritin, µg/l, female^b^ | 0 wk | 57.8 ± 41.0 | 47.1 ± 39.8 | 61.4 ± 53.9 |  |
|  | 12 wk | 45.8 ± 35.7^c^ | 37.2 ± 29.5 | 48.1 ± 43.3 | 0.659 |
|  |  |  |  |  |  |
| Plasma ferritin, µg/l, male^b^ | 0 wk | 87.2 ± 25.2 | 127.3 ± 101.9 | 141.2 ± 76.4 |  |
|  | 12 wk | 82.0 ± 29.8 | 111.3 ± 85.1 | 114.9 ± 57.1 | 0.685 |
|  |  |  |  |  |  |
| Plasma TfR, mg/l, female^b^ | 0 wk | 3.2 ± 0.9 | 3.4 ± 0.7 | 3.3 ± 1.0 |  |
|  | 12 wk | 3.4 ± 0.8 | 3.6 ± 0.8 | 3.4 ± 0.8 | 0.095 |
|  |  |  |  |  |  |
| Plasma TfR, mg/l, male^b^ | 0 wk | 3.5 ± 0.4 | 2.9 ± 0.7^d^ | 3.1 ± 0.5^d^ |  |
|  | 12 wk | 3.6 ± 0.4 | 3.3 ± 0.4 | 3.1 ± 0.4 | 0.112 |
|  |  |  |  |  |  |
| Haemoglobin, g/l, female^b^ | 0 wk | 132.4 ± 6.9 | 128.4 ± 8.8 | 132.4 ± 8.9 |  |
|  | 12 wk | 132.2 ± 7.0 | 127.2 ± 9.2 | 131.2 ± 8.2 | 0.346 |
|  |  |  |  |  |  |
| Haemoglobin, g/l, male^b^ | 0 wk | 149.7 ± 7.2 | 143.3 ± 8.3 | 147.7 ± 8.1 |  |
|  | 12 wk | 146.8 ± 6.6 | 145.8 ± 8.1 | 145.0 ± 7.8 | 0.399 |
|  |  |  |  |  |  |
| Serum folate, nmol/l | 0 wk | 19.5 ± 6.9 | 17.0 ± 6.1 | 17.7 ± 5.6 |  |
|  | 12 wk | 15.8 ± 5.3 | 15.3 ± 4.7 | 15.2 ± 4.6 | 0.819 |
|  |  |  |  |  |  |

^a^Values are means ± SDs, ANIMAL, a diet containing 70% animal and 30% plant protein sources of total protein intake; 50/50, a diet containing equal proportions (50:50) of animal and plant protein sources of total protein intake; PLANT, a diet containing 30% animal and 70% plant protein sources of total protein intake. *P-*values from ANCOVA, adjusted for baseline with Bonferroni correction.

**P-*values from ANOVA, with Bonferroni correction.

^b^indicators of iron status separately for female and male participants, for females ANIMAL *n* = 37, 50/50 *n* = 36 and PLANT *n* = 34, for males ANIMAL *n* = 9, 50/50 *n* = 10 and PLANT *n* = 10

^c^*n* = 36

^d^*n* = 9

^†^post hoc comparison (Bonferroni correction) between ANIMAL and 50/50, *P*<0.05

^‡^post hoc comparison (Bonferroni correction) between ANIMAL and PLANT, *P*<0.05

^ͳ^post hoc comparison (Bonferroni correction) between 50/50 and PLANT, *P*<0.05

na = not available

Abbreviations: HoloTC, holotranscobalamin II; TfR, transerrin receptor; U–I, 24-hour urinary iodine excretion

**Table S6** Multivariable analysis for iron status indicators (ferritin, transferrin receptor, and haemoglobin) at end point of the intervention among healthy adults (*n* = 136) who consumed intervention diets differing in animal and plant protein levels for 12 weeks^a^

| Pillai | approx F | num Df | den DF | *P*-value |
| --- | --- | --- | --- | --- |
| 0.1292756 | 1.429778 | 12 | 381 | 0.1495595 |
